# New insights into the pathogenesis and prevention of tuberous sclerosis-associated neuropsychiatric disorders (TAND)

**DOI:** 10.12688/f1000research.11110.1

**Published:** 2017-06-09

**Authors:** Tanjala T. Gipson, Michael V. Johnston

**Affiliations:** 1 Boling Center for Developmental Disabilities, LeBonheur Children’s Hospital, University of Tennessee Health Sciences Center, Memphis, TN, USA; 2 Kennedy Krieger Institute, Johns Hopkins University School of Medicine, Baltimore, MD, USA

**Keywords:** TSC1, TSC2, neuropsychiatric disorder, tuberous sclerosis complex

## Abstract

Tuberous sclerosis complex (TSC) is a multi-system disorder resulting from mutations in either the *TSC1* or *TSC2 *genes leading to hyperactivation of mechanistic target of rapamycin (mTOR) signaling. TSC is commonly associated with autism (61%), intellectual disability (45%), and behavioral, psychiatric, intellectual, academic, neuropsychological, and psychosocial difficulties that are collectively referred to as TSC-associated neuropsychiatric disorders (TAND). More than 90% of children with TSC have epilepsy, including infantile spasms, and early onset of seizures, especially infantile spasms, is associated with greater impairment in intellectual development compared with individuals with TSC without seizures. Development of the mTOR inhibitors everolimus and sirolimus has led to considerable progress in the treatment of renal angiomyolipomata, pulmonary lymphangioleiomyomatosis, and subependymal giant cell astrocytomas in the brain. However, similar therapeutic progress is needed in the treatment of TAND.

Tuberous sclerosis complex (TSC) affects 1 out of 6,000 live births and is the second most common neurocutaneous disease (neurofibromatosis is the most common) ^1^. The skin manifestations of TSC, including facial angiofibromas, periungual fibromas, hypomelanotic macules formerly known as “ash leaf spots”, and areas of thickened leathery skin (shagreen patches), were recognized clinically long before the pathogenesis was identified. TSC causes multi-system involvement, including benign tumors in the brain, kidneys, heart, eyes, lungs, and skin as well as seizures, intellectual disability, autism spectrum disorders, and other neuropsychiatric disorders. It is inherited in an autosomal dominant pattern, but the rate of spontaneous mutations is high ^1^. The neurobiology of TSC has been linked to mutations in the *TSC1* or *TSC2* genes leading to dysfunction of the TSC1 or TSC2 proteins, which form a complex that usually inhibits mechanistic target of rapamycin (mTOR) ^2, 3^. These proteins form a complex that integrates the input from upstream cellular pathways, including insulin, growth factors (such as insulin-like growth factor-1 and brain-derived neurotrophic factor), and amino acids. The mTOR pathway is a central regulator of mammalian metabolism and physiology and normally senses cellular nutrient, oxygen, and energy levels ^4^. Dis-inhibition of this pathway caused by loss-of-function mutations in *TSC1* or *TSC2* leads to generally benign overgrowth or tumors in brain, kidney, skin, eye, and other organs that are the clinical hallmarks of the disorder ^3^. In addition to TSC, alterations in mTOR have been reported in a number of other genetic neurologic disorders, including neurofibromatosis 1, fragile X syndrome, Proteus syndrome, and phosphatase and tensin homolog (PTEN) mutations in Cowden syndrome, Lhermitte-Duclos syndrome, and related disorders ^3^. PTEN mutation disorders are usually associated with macrocephaly and may be associated with cancer.

A specific combination of major and minor features of TSC is required to make a clinical diagnosis of the disorder ( Table 1) ^5^. An identified genetic mutation in either *TSC1* or *TSC2* is also an independent criterion for diagnosis. Epilepsy (90%) ^6– 13^, intellectual disability (45%) ^14– 18^, and autism (61%) ^19– 33^ are prominent features of TSC but are relatively non-specific and are not diagnostic. Autism, intellectual disability, and related conditions are classified as TSC-associated neuropsychiatric disorders (TAND) ^34^.

**Table 1.** Major and minor features required to make a clinical diagnosis of tuberous sclerosis complex.

Major features	Minor features
1. Hypomelanotic macules (≥3 at least 5 mm in diameter)	1. “Confetti” skin lesions
2. Angiofibromas (≥3) or fibrous cephalic plaque	2. Dental enamel pits (>3)
3. Ungual fibromas (≥2)	3. Intraoral fibromas (≥2)
4. Shagreen patch	4. Retinal achromatic patch
5. Multiple retinal hamartomas	5. Multiple renal cysts
6. Cortical dysplasias ^a^	6. Nonrenal hamartomas
7. Subependymal nodules	
8. Subependymal giant cell astrocytoma	
9. Cardiac rhabdomyoma	
10. Lymphangioleiomyomatosis ^b^	
11. Angiomyolipomas (>2) ^b^	

^a^Definite diagnosis: Two major features or one major feature with at least two minor features. Possible diagnosis: Either one major feature or at least two minor features. Cortical dysplasia includes tubers and cerebral white matter radial migration lines. ^b^A combination of the two major clinical features (lymphangioleiomyomatosis and angiomyolipomas) without other features does not meet criteria for a definite diagnosis.

Two drugs that inhibit mTOR, sirolimus and everolimus, which were originally developed as immunosuppressive and anti-cancer agents, have recently been applied to treat the manifestations of TSC. These drugs are now US Food and Drug Administration (FDA)-approved for TSC-associated subependymal giant cell astrocytomas (SEGAs), renal angiomyolipomata, and pulmonary lymphangioleiomyomatosis ^35– 38^. Deficiency of the major inhibitory neurotransmitter amino acid GABA (gamma-aminobutyric acid) has been linked to the high incidence of seizures in TSC ^39, 40^, and the anticonvulsant drug vigabatrin, an irreversible inhibitor of GABA transaminase, which degrades GABA, has been shown to be effective for treatment of infantile spasms and refractory complex partial seizures associated with TSC ^41, 42^. European practice guidelines recommend it as a first line for treatment of seizures in infants with TSC ^43^, and it is considered as neurobiologically targeted treatment for TSC.

## Treatment of seizures in patients with tuberous sclerosis complex

Several recent studies have reported beneficial effects of everolimus for epilepsy in patients with TSC. Krueger *et al*. ^44^ studied the effect of everolimus in children older than 2 years of age with confirmed TSC and medically refractory epilepsy averaging two or more seizures a week in the months prior to enrollment. These patients failed to respond to trials of two other anticonvulsants. Fourteen of 18 patients who completed the study over 48 months were reported to have a 48% reduction in seizures with few side effects. Samueli *et al*. ^45^ reported on the effect of everolimus in 15 children with TSC less than 18 years of age who had focal seizures, tonic clonic-seizures, or drop attacks (or a combination of these); the authors found that 80% of patients reported fewer seizures and that 58% were seizure-free at a median follow-up of 22 months. French *et al*. ^46^ reported on a placebo controlled trial of 366 patients (age range of 2 to 65 years) who were given everolimus as an add-on therapy for treatment-resistant focal onset seizures associated with TSC (EXIST-3); the authors found response rates of 29% in patients given a low dose, 40% in patients given a higher dose, and 15% in a placebo control group. Adverse drug reactions led to discontinuation of medication in 2%. These trials indicate that mTOR inhibition shows promise for improving seizure control in patients with TSC.

## Spectrum of tuberous sclerosis complex-associated neuropsychiatric disorders

TAND is a term that encompasses the multi-faceted nature of neurodevelopmental disabilities seen in patients with TSC, including behavioral, psychiatric, intellectual, academic, neuropsychological, and psychosocial difficulties ^47– 49^. Behavioral manifestations may be relatively mild such as poor eye contact or more severe disorders on the autism spectrum, including self-injury (10%) and aggression (45%) ^49– 51^. Anxiety, depression, attention-deficit/hyperactivity disorder, autism spectrum disorder, and other psychiatric concerns are also common among patients with TSC ^13, 52– 55^. It is estimated that as many as 50% may be impacted by some level of intellectual disability ^13, 52– 56^. Academic difficulties in the absence of intellectual disability have also been observed in 30% of individuals with TSC ^56^. Specific neuropsychological deficits, such as impaired working memory, may also be problematic ^55^. The concept of TAND also encompasses the psychosocial impact of the individual’s condition on the rest of the family ^57^. To enable physicians and other medical professionals to screen for these concerns, the TAND checklist has been created ^34^.

## Treatment of tuberous sclerosis-associated neuropsychiatric disorder

There are no medications approved by the FDA for treatment of TAND, and more progress is needed in this area. Several studies of genetic mouse models of TSC suggest that mTOR inhibitors can reverse deficits in synaptic plasticity, neurobehavior, social impairment, and repetitive behaviors ^3^. In an open-label phase II trial to evaluate the efficacy and safety of rapamycin in adults with angiomyolipomata and TSC, seven out of eight patients had improvement in neurocognitive deficits, including list learning and story recall ^58^. Two studies are in progress to address the treatment of TAND ^46^. The RAD001 Neurocognition Trial ( www.ClinicalTrials.gov/ct2/show/NCT01289912) is a phase II randomized trial of everolimus in children and young adults ages 6 to 21 years who are being followed with neuropsychological testing. The phase III EXIST-3 trial (NCT01713946) is evaluating the effects of everolimus by using the Vineland Adaptive Behavior Scales, Second Edition ^59^. In a young man with TSC, a SEGA, epilepsy, aggression, and self-injurious behavior, treatment with everolimus resulted in size reduction of his SEGA, reduction in self-injurious behavior, and diminished seizure frequency ^60^. Discovering the potential role for neurobiologically targeted medications for behavior in TSC is the basis of our ongoing research program.

## Early treatment of infantile spasms may salvage cognitive development

The inter-relationship between infantile spasms and TAND in tuberous sclerosis is an interesting one that may hold clues to the pathways by which TSC can cause autism and intellectual disabilities. Approximately 30% of patients with TSC present in infancy with infantile spasms, a severe form of epilepsy associated with a hypsarrhthymia pattern on electroencephalography. These patients are at much greater risk for severe autism, intellectual disability, and behavioral disorders compared with TSC patients who do not have infantile spasms. On the other hand, patients with idiopathic infantile spasms are at much lower risk for TAND than are patients with TSC. Early treatment with vigabatrin appears to be protective against severe TAND. For example, one study of infants with TSC compared (a) initiation of treatment with vigabatrin at the onset of electroencephalogram (EEG) abnormalities on anticipatory recordings determined before seizures were witnessed with (b) treatment initiation upon the onset of witnessed seizures ^61^. No cases of severe/profound intellectual disability were detected among those with the EEG-based treatment ^61, 62^. A second study, designed to address the question of whether an EEG could be used as a biomarker in infants with TSC, validated its use in this regard ^63^. Bolton *et al*. ^16^ reported that *TSC2* mutations were also associated with significantly higher cortical tuber count than *TSC1* mutations, and the tuber count was directly related to earlier age and onset of seizures. In turn, the severity of epilepsy was strongly associated with the degree of intellectual impairment. Structural equation modeling, a multivariate statistical analysis technique that combines factor and multiple regression analysis to analyze structural relationships, supported a causal pathway from genetic abnormality to tuber count to epilepsy, including infantile spasms, to severity of intellectual outcome ^64^. Humphrey *et al*. reported that there is a dose-dependent impairment of intellectual development following exposure to infantile spasms in children with TSC ^64^. Recently, it was reported that hormonal therapy with either adrenocorticotropic hormone or prednisolone plus vigabatrin is significantly more effective than hormonal therapy alone at stopping infantile spasms in infants without TSC ^65, 66^. Therefore, evaluating combination therapy could be useful in patients with TSC and spasms as early termination of spasms is critically important to prevent additional injury.

## Summary

Tuberous sclerosis is a genetic multi-system disease that can have a major impact on the developing brain to cause seizures and an array of severe neuropsychiatric disorders, including severe intellectual disability and autism spectrum disorders. Recognition of the role of mutations in the *TSC1* and *TSC2* genes and the mTOR signaling pathways has led to the use of mTOR inhibitors to arrest or lessen the impact of the disorder. These medications have had a major impact in the brain and may also have an anti-epileptic effect. The concept of TAND has led to greater recognition of a host of disorders that can cause a great deal of impairment. The development of a common terminology for TAND to address the varying levels of involvement and the creation of the TAND checklist to aid clinicians and researchers in screening for these concerns provide a foundation for uniformity. Recognition that early onset of seizures, especially infantile spasms, are common in infants with TSC and that early onset of infantile spasms and associated hypsarrhythmia may have a malignant effect on brain development in infants with TSC has stimulated the search for ways to anticipate the onset of infantile spasms before they become apparent as seizures. Recent data suggesting that hormonal therapy combined with vigabatrin may stop spasms more quickly may have important benefits for patients with TSC. This information, combined with an understanding of the genetics and neurobiology of TSC, is leading to a deeper understanding of pathogenesis and possibly better therapies.
